# Predictive role of galectin-1 and integrin α5β1 in cisplatin-based neoadjuvant chemotherapy of bulky squamous cervical cancer

**DOI:** 10.1042/BSR20170958

**Published:** 2017-09-28

**Authors:** Haiyan Zhu, Aixue Chen, Saisai Li, Xuejiao Tao, Bo Sheng, Mandika Chetry, Xueqiong Zhu

**Affiliations:** 1 Department of Obstetrics and Gynecology, The Second Affiliated Hospital of Wenzhou Medical University, Wenzhou 325027, China; 2 Department of Obstetrics and Gynecology, The Second Hospital of Cangnan People’s Hospital, Wenzhou 325027, China

**Keywords:** Galectin-1, integrin α5β1, immunohistochemistry, neoadjuvant chemotherapy, squamous cervical cancer

## Abstract

Although galectin-1 and integrin α5β1 confer chemoresistance to certain types of cancer, whether their expression predicts the response to cisplatin-based neoadjuvant chemotherapy (NACT) in squamous cervical cancer remains unclear. Paired tumor samples (pre- and post-chemotherapy) were obtained from 35 bulky squamous cervical cancer patients treated with cisplatin-based NACT and radical hysterectomy at our hospital between January 2007 and August 2014. The expression of galectin-1 and integrin α5β1 in tumor cells and stromal cells was analyzed by immunohistochemistry. The correlation between galectin-1/integrin α5β1 and apoptosis-associated markers was investigated by using the The Cancer Genome Atlas (TCGA) RNA-sequencing data. Seventeen patients were identified as chemotherapy responders and 18 as non-responders. Galectin-1 and integrin α5β1-positive immunostaining was more frequently observed in stromal cells than its in tumor cells. The expression of galectin-1 and integrin α5β1 in stromal and tumor cells was significantly down-regulated in postchemotherapy cervical cancer tissues. High levels of galectin-1 and integrin α5β1 in stromal were associated with a negative chemotherapy response in squamous cervical cancer patients treated with cisplatin-based NACT. Additionally, the expression of galectin-1 and integrin α5 correlated negatively with caspase 3/caspase 8 by using the TCGA RNA-sequencing data. Galectin-1 and integrin α5β1 expression in stromal may serve as a prediction of the responses to cisplatin-based NACT for patients with bulky squamous cervical cancer. Galectin-1 and integrin α5β1 may be implicated in the development of chemoresistance in cervical cancer via suppressing apoptosis.

## Introduction

Cervical cancer is the second most commonly diagnosed cancer and the third leading cause of cancer deaths amongst females in less developed countries, with an estimated 527600 new cases and 265700 deaths worldwide in 2012 [1]. Even in the United States, where the prevention of cervical cancer by Pap smear based screening and treatment programs have been largely successful, approximately 12900 new cases and 4100 deaths from cervical cancer were estimated for 2015 [2]. Specifically, the prognosis of locally advanced cervical cancer (tumor size ≥4 cm) was very poor after conventional treatments, with a 5-year overall survival rate of approximately 40% [3]. Recently, neoadjuvant chemotherapy (NACT) prior to surgery has been widely used for bulky or locally advanced cervical cancer. This new therapeutic strategy can shrink the tumor volume, control micrometastases and improve the safety and integrity of surgery [3]. Nevertheless, the prognosis of NACT-refractory patients would become worse due to the delay in curative treatment [4]. Currently, there is no method to reliably predict tumor responses to NACT prior to the therapy. Therefore, it is of great clinical value to identify and develop effective biomarkers that can predict the resistance to NACT and thus to assess the treatment options of individual patients.

In our previous study, we have identified 16 proteins, including galectin-1, which were down-regulated in squamous cervical cancer tissue after NACT relative to the level before chemotherapy by using Proteomic Profiling [5]. Galectin-1, belonging to the β-galactoside-binding lectin family, is well known to be involved in a wide range of physiologic processes such as cell transformation, cell proliferation, angiogenesis, cell adhesion and invasiveness, and immunosuppression [6]. Indeed, accumulating studies have explored its pivotal role in the carcinogenesis of cervix. Kohrenhagen et al. [7] reported that galectin-1 expression on stromal cells increased with the histopathologic grade of cervical tissues. High galectin-1 expression in peritumoral stroma was significantly correlated with the depth of invasion in the cervix and lymph node metastasis [8]. Galectin-1 inhibition by siRNA or galectin-1 antibody suppressed the growth of cervical cancer both *in vitro* and *in vivo* [8,9]. Furthermore, galectin-1 is an independent prognostic factor for local recurrence and survival in stage I–II cervical cancer patients undergoing definitive radiation therapy [10].

Recent evidence indicates that galectin-1 exerts a potent role in the development of chemoresistance. Suppression of galectin-1 can sensitize lung cancer cells to cisplatin [11,12], while increase the expression of galectin-1 in epithelial ovarian cells can enhance their resistance to cisplatin [13]. However, whether galectin-1 expression correlates with chemoresistance in squamous cervical cancer patients is currently unknown.

Integrins are heterodimeric transmembrance adhesion receptors, which contain two different chains, α and β subunits [14]. Integrins mediate a wide variety of cell–cell and cell–matrix interactions that lead to cell migration, proliferation, differentiation, and survival. Integrin α5 primarily combines with integrin β1 subunit to form α5β1 heterodimer [15]. Integrin α5β1 is one of the integrin family members and could play a role in cell survival and chemoresistance in certain types of cancer [16–18]. With respect to human cervical cancer, integrin α5β1 has been detected overexpressed in cervical cancer tissues, and high levels of integrin α5β1 were correlated with poor differentiation, lymph node metastasis, recurrence as well as a poor prognosis in cervical cancer [19]. However, none of the studies has evaluated the possible role of integrin α5β1 in chemotherapy responses amongst cervical cancer patients.

In the present study, we first examined the correlation between the expression of galectin-1 and integrin α5β1 and the responses to cisplatin-based NACT in patients with stage IB2 or IIA2 squamous cervical cancer, and then explored their potential mechanism(s) using the The Cancer Genome Atlas (TCGA) and GEO RNA-sequencing data.

## Materials and methods

### Ethics statement

The present study was approved by the Ethical Committee of the Second Affiliated Hospital of Wenzhou Medical University and conducted according to the Helsinki Declaration. Written informed consents were granted from all the subjects at the time of enrollment.

### Patients and tissue specimens

Patients with IB2 or IIA2 (bulky, primary tumor >4 cm in diameter) squamous cervical cancer at the Second Affiliated Hospital of Wenzhou Medical University, Wenzhou, China, between January 2007 and August 2014, were recruited for a pilot study aimed to identify predictive biomarkers for responses to NACT. Patients were included if they met the following criteria: (i) Patients had confirmed diagnosis of squamous cervical cancer by pathology. (ii) The lesions in non-pregnant patients who had normal bone marrow, liver, and kidney functions were measured by physical examination, transvaginal ultrasound, and MRI. Only individuals with bulky stage IB2 or IIA2 cervical cancer (primary tumor >4 cm in diameter) were included. (iii) Patients who did not complete the planned cycles of NACT were excluded. (iv) Patients receiving chemotherapy, immunotherapy, hormonal therapy, or radiotherapy before the specimen collection were excluded. Finally, a total of 35 subjects were enrolled with a median age of 46 years (range: 25–63 years).

Paired tumor samples from each patient were obtained during cervical biopsy (prechemotherapy) or surgery (postchemotherapy). The biopsies from each patient were put into 4% paraformaldehyde in 0.1 M phosphate buffer (pH 7.4), embedded in paraffin, and cut into 4-μm sections. Then, hematoxylin and eosin stained slides of the cervical tumor were reviewed for confirmation of pathological features, as well as to select suitable tissue blocks for immunohistochemical analysis.

### NACT and therapeutic effect evaluation

All eligible patients received one or two courses of cisplatin-based NACT, as previously described [20]: cisplatin, 60 mg/m^2^ on day 1; 5-fluorouracil, 750 mg/m^2^ on day 1; mitomycin, 8 mg/m^2^ on day 1 for one or two courses, every 28 days. All these chemotherapeutics were administrated via uterine artery injection. The chemotherapy response was evaluated by measuring the tumor’s two dimensions (the longest diameter and its perpendicular diameter) with MRI or other radiographic means. The chemotherapy response was determined 2 weeks after completion of NACT, according to the WHO criteria [21]. Complete response (CR) was defined as the complete remission of the tumor. Partial response (PR) was defined as at least a 50% decrease in the tumor volume. Stable disease (SD) meant a steady state or a response less than 50%, and progressive disease (PD) was defined as an unequivocal increase in at least 25% of the tumor volume. The patients with CR or PR were defined as chemotherapy responders, while the patients with SD or PD were deemed as chemotherapy non-responders. All patients were treated with radial hysterectomy and bilateral pelvic lymphadenectomy 2–3 weeks after completion of the NACT regimen as described previously [4].

### Immunohistochemistry

Immunohistochemical staining for galectin-1 and integrin α5β1 was performed in all the cases. Biopsy was embedded in paraffin, cut into 4-μm sections, and then mounted on to poly-L-lysine-coated slides. Briefly, after deparaffinization in xylenes and rehydration through graded ethanol solutions, sections were boiled in 10 μmol/l citric buffer solution (pH 6.0) in a microwave oven for 10 min, followed by incubation with 3% hydrogen peroxide in methanol to suppress the endogenous peroxidase activity and overnight incubation with primary antibodies at 4°C. The following primary antibodies and corresponding dilutions were used: galectin-1 (Abcam, U.S.A.; 1:200) and integrin α5β1 (Millipore, U.S.A.; 1:200). Subsequently, the sections were incubated with prediluted biotinylated goat anti-mouse or goat anti-rabbit secondary antibody (Santa Cruz Technology, U.S.A.) for 2 h at room temperature, followed by further incubation with 3,3-diaminobenzidine tetrahydrochloride (DAB). Finally, the slides were counterstained with Hematoxylin and mounted in an aqueous mounting medium. Appropriate positive and negative controls were stained in parallel. For negative controls, primary antibodies were replaced with PBS solution. Human cervical cancer tissues were used as a positive control.

### Evaluation of immunoreactivity

Galectin-1 protein was detected primarily in cytoplasm and partially in cytomembrane. Integrin α5β1 protein was observed mainly in cytoplasm and partially in nucleus. Additionally, galectin-1 and integrin α5β1 proteins were detected both in the tumor cells and the stroma cells of squamous cervical cancer samples. To assess all positively expressing cells, the amount of galectin-1 and integrin α5β1 was analyzed both in tumor cells and stromal cells. Staining evaluation was assessed by two independent observers, who were blinded to the clinical outcomes. Expression of the two markers was determined by an individual labeling score considering percent and staining intensity of positive cells [22]. Intensity of stained cells was graded semiquantitatively into four levels as following: 0 (no staining); 1 point (weak staining : light yellow); 2 points (moderate staining : yellow brown), and 3 points (strong staining:  brown). The percentage was scored as following: 0 (0–5%), 1 point (6–24%), 2 points (25–49%), 3 points (50–74%), and 4 points (75–100%). Intensity and fraction of positive cell scores were multiplied for each marker, and thus, we got the immunoreactive score.

### Bioinformatics analysis

Three publicly available datasets, including TCGA database, GSE6791 and GSE9750 were used in the current study. Briefly, RNA-sequencing data, including 309 cervical samples were downloaded from TCGA database (https://gdc-portal.nci.nih.gov/) and Gene Expression Omnibus (GEO) database as referenced above were downloaded from https://www.ncbi.nlm.nih.gov/gds/.

Integrin α5 primarily combines with integrin β1 subunit to form α5β1 heterodimer, therefore, we will analyze integrin α5 and integrin β1 genes, respectively. There are only three cases of cervical normal tissues in TCGA database, therefore, the mRNA expression of galectin-1 and integrin α5β1 in cervical cancer tissues and normal tissues was determined through analysis of GEO databases. The correlation between galectin-1/integrin α5β1 and apoptosis-associated markers (caspase 3 and caspase 8) was investigated by using the TCGA RNA-sequencing data.

### Statistical analysis

The Kolmogorov–Smirnov test of normality was applied. Continuous variables were presented as means ± S.D. and non-normally distributed variables were presented as median (P25– P75). Since the patient profile between the NACT responsive and non-responsive groups displayed a normal distribution, a Student’s *t* test (age and tumor size) or chi-square test (FIGO stage and histological grade) was used for analysis. Protein expression between the two groups displayed a non-normal distribution and was evaluated by Wilcoxon test (comparison between pre- and postchemotherapy groups) or Mann–Whitney U test (comparison between chemotherapy response and non-response groups). The software of SPSS 16.0 (SPSS Inc, IL) was used for statistical analysis. A *P-*value less than 0.05 was considered statistically significant.

## Results

### Patient characteristics

Clinical and pathological characteristics of 35 women with squamous cervical cancer are summarized in Table 1. A CR and a PR were obtained in 5 and 12 patients, respectively, with an overall optimal response of 48.6%. Thirteen patients (37.1%) had an SD and other five patients (14.3%) showed PD. There were no significant differences between the chemotherapy responders and non-responders in age at diagnosis, the disease’s FIGO stage, primary tumor size, and differentiation (*P*>0.05) (Table 1).

**Table 1:** Clinical characteristics of cervical cancer patients between NACT responders and non-responders

Parameter	Responders (*n*=17)	Non-responders (*n*=18)	*P*
Median age (years)	47 ± 8	44 ± 9	NS*
FIGO stage			NS*
IB2	8	11	
IIA2	9	7	
Differentiation			
G1	6	7	
G2	10	10	
G3	1	1	
Primary tumor size (cm)	4.53 ± 1.24	4.00 ± 1.41	NS*

* *P*>0.05.

NS: no significance

### Expression of galectin-1 and integrin α5β1 status

**Figure 1:**
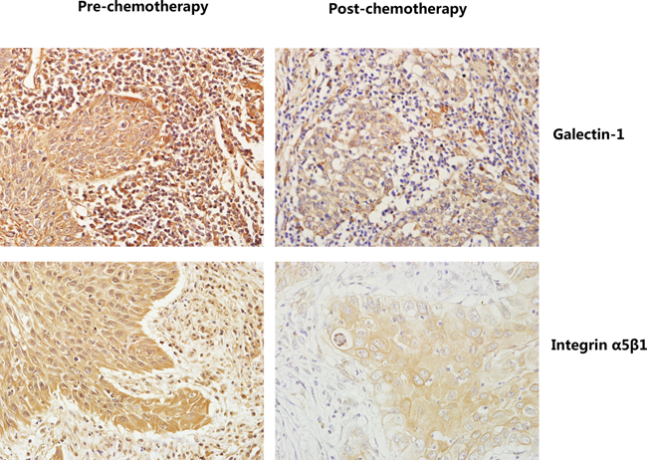
The expression of galectin-1 and integrin α5β1 in paired samples of squamous cervical cancer as compared with corresponding prechemotherapy and postchemotherapy (SP staining, ×400). Galectin-1 and integrin α5β1 proteins were detected both in the tumor cells and the stroma cells of squamous cervical cancer samples. Galectin-1 protein primarily localized in the cytoplasm and partially in the cytomembrane, while integrin α5β1 protein mainly expressed in the cytoplasm and partially in nuclei of these cells.

The expression of galectin-1 and integrin α5β1 in 35 matched primary squamous cervical cancer biopsies before and after cisplatin-based NACT were evaluated using immunohistochemistry. As shown in Figure 1, galectin-1 and integrin α5β1 proteins were detected both in the tumor cells and in the stroma cells of squamous cervical cancer samples. To assess all the positively expressing cells, the amount of galectin-1 and integrin α5β1 was analyzed both in tumor cells and stromal cells. Galectin-1 and integrin α5β1 expression were detected in stroma amongst all the 70 samples. However, galectin-1 and integrin α5β1 protein expressed in tumor cells only in 82.86% (58/70) and 85.71% samples (60/70), respectively. Additionally, galectin-1 protein primarily localized in the cytoplasm and partially in the cytomembrane, while integrin α5β1 protein mainly expressed in the cytoplasm and partially in nucleus of these cells.

### Proteins expression in cervical cancers pre- and postchemotherapy

Initially, the effect of chemotherapy on the expression of galectin-1 and integrin α5β1 in squamous cervical cancer samples was evaluated. Prechemotherapy cervical cancer tissues consistently showed moderate or intense positive staining of galectin-1 and integrin α5β1 in tumor cells, while postchemotherapy tissues consistently showed weak or moderate positive staining in tumor cells (Figure 1). Using a Wilcoxon test, the expression of galectin-1 and integrin α5β1 in tumor cells was significantly decreased in postchemotherapy samples compared with prechemotherapy tissue samples, respectively (*P*<0.05) (Table 2). With regard to proteins’ expression in stromal cells, galectin-1 and integrin α5β1 protein expression were also markedly down-regulated after cisplatin-based NACT when compared with their matched prechemotherapy tissues, consistent with their expression in tumor cells (Figure 1, Table 2).

**Table 2:** The expression of galectin-1 and integrin α5β1 in cancer cells and stromal cells of pre- and postchemotherapy cervical squamous cancer

	*n*	Galectin-1		Integrin α5β1	
		Cancer cells	Stromal cells	Cancer cells	Stromal cells
Prechemotherapy	35	8.0 (4.00–8.00)	9.0 (7.00–12.00)	8.0 (6.50–11.00)	8.0 (6.00–11.25)
Postchemotherapy	35	4.0 (2.00–8.00)	6.0 (4.00–6.00)	4.0 (3.00–8.00)	2.0 (2.00–4.00)
Z		–3.108	–4.228	–3.617	–3.484
*P*		0.002*	0.00*	0.00*	0.00*

Data are expressed as median (P25–P75), *P<0.05.

Collectively, chemotherapy down-regulated the expression of galectin-1 and integrin α5β1 both in tumor cells and stromal cells of squamous cervical cancer, suggesting galectin-1 and integrin α5β1 protein were involved in cervical tumor response to chemotherapy.

### Proteins expression in cervical cancers between chemotherapy response and non-response groups

Then, the involvement of galectin-1 and integrin α5β1 proteins in predicting responses to NACT was further investigated. The expression of galectin-1 and integrin α5β1 in prechemotherapy cervical cancer tissues between chemotherapy responders and non-responders was analyzed by immunohistochemistry.

**Figure 2:**
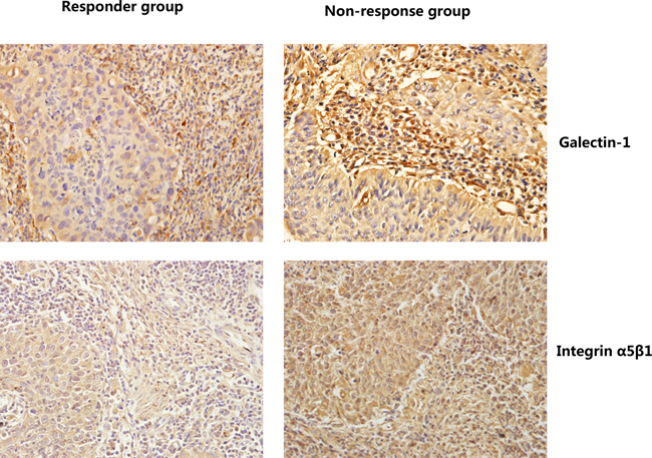
The expression of galectin-1 and integrin α5β1 in cervical squamous cancer cells and stromal cells in the chemotherapy response group and the non-response group (SP staining, ×400) The expression of galectin-1 and integrin α5β1 in stromal was much lower in the response group than that in the non-response group.

As shown in Table 3, the expression of galectin-1 and integrin α5β1 in stromal cells was statistically lower in the response group than that in the non-response group, suggesting that patients with high levels of galectin-1 and integrin α5β1 in stromal were more resistant to cisplatin-based NACT than those with low protein expression (*P*<0.05, respectively) (Figure 2, Table 3). Nevertheless, the expression of galectin-1 and integrin α5β1 in tumor cells had no statistical difference between these two groups (Figure 2, Table 3). Thus, we suggested that the expression of galectin-1 and integrin α5β1 protein in stromal cells but not in tumor cells was associated with responses to NACT among squamous cervical cancer patients.

**Table 3:** The expression of galectin-1 and integrin α5β1 in cancer cells and stromal cells of prechemotherapy cervical cancer between chemotherapy response and non-response groups

	*n*	Galectin-1		Integrin α5β1	
		Cancer cells	Stromal cells	Cancer cells	Stromal cells
Responders	17	8.0 (4.00–8.00)	8.0 (6.00–9.00)	8.0 (6.00–8.00)	6.0 (4.00–8.00)
Non-responders	18	8.0 (4.00–8.00)	12.0 (8.00–12.00)	8.0 (8.00–12.00)	8.0 (8.00–12.00)
Z		–0.342	–2.327	–1.449	–2.795
P		0.732	0.020*	0.147	0.005*

Data are expressed as median (P25–P75), *P<0.05.

### The correlation between galectin-1/integrin α5β1 and apoptosis-associated markers in human cervical cancer tissues

**Figure 3:**
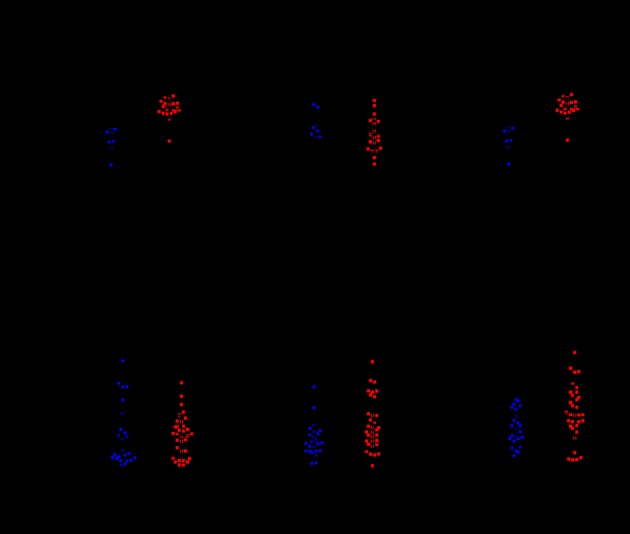
Galectin-1 and integrin α5β1 are up-regulated in human cervical cancer tissues (**a**) GSE6791 data showing the expression of galectin-1, integrin α5, and integrin β1 in normal cervical tissues compared with cervical cancer. (**b**) GSE9750 data showing the expression of galectin-1, integrin α5, and integrin β1 in normal cervical tissues compared with cervical cancer.

To determine the clinical relevance of the expression of galectin-1 and integrin α5β1, we first analyzed the mRNA expression of galectin-1 and integrin α5β1 using the GEO RNA-sequencing data. As shown in Figure 3, the mRNA levels of galectin-1, integrin α5, and integrin β1 were much higher in cervical cancer tissues than that in normal cervical tissues.

Cisplatin-induced apoptosis was essential for the anticancer effect of cisplatin [23], we then explored the correlation between galectin-1/integrin α5β1 and apoptosis-associated markers. Since the sample size in GEO database was not large enough (n=28 and n=66, respectively), we then analyzed their correlation by using the TCGA RNA-sequencing data (n=309). Interestingly, a significant negative correlation was detected between galectin-1/integrin α5 and caspase 3/caspase 8 (Table 4).

**Table 4:** The relevance between galectin-1/integrin α5/integrin β1 and apoptosis associated-markers in cervical cancer

Group	*n*	Caspase 3		Caspase 8	
		r	*P*	r	*P*
Galectin-1	309	–0.296	0.000*	-0.211	0.000*
Integrin α5	309	–0.124	0.029*	-0.112	0.05*
Integrin β1	309	0.035	0.541	0.101	0.077

*P<0.05; n, numbers of patients.

## Discussion

In the current study, we reported that galectin-1 and integrin α5β1 expression in tumor cells and stromal cells were significantly down-regulated after NACT in patients with squamous cervical cancer. The expression of galectin-1 and integrin α5β1 in stromal was much higher in patients with poor response to cisplatin-based NACT than that in the response group. These findings suggest that galectin-1 and integrin α5β1 may serve as potential predictive biomarkers to cisplatin-based NACT responses for patients with squamous cervical cancer. To our knowledge, this is the first study to investigate the predictive role of galectin-1 and integrin α5β1 in chemoresistance amongst squamous cervical cancer patients.

Galectin-1 is a β-galactoside-binding protein that is abundantly secreted by almost all the types of malignant tumor cells [24]. It is involved in numerous physiologic processes including cell transformation, cell proliferation, angiogenesis, cell adhesion and invasiveness, and immunosuppression [6,24]. Galectin-1 has been widely reported to be overexpressed in cervical cancer cell lines and cervical cancer samples [7,8,25]. In agreement with previous data, we found that galectin-1 mRNA was much higher in cervical cancer samples compared with normal cervical tissues. Our current study detected galectin-1 expressed both in tumor cells and stromal cells, moreover, galectin-1 accumulated more frequently in stromal cells than its in tumor cells of cervical cancer, which is consistent with previous immunohistochemical studies [7,8,25]. In addition, galectin-1 has been detected in the cytoplasm, cytomembrane, and nucleus, mainly located in the cytoplasm [25,26], which was further confirmed by the current study.

In addition to its role in tumorigenesis, the association between galectin-1 expression and chemoresistance was investigated in several types of cancer more recently. Galectin-1 expression was found to be up-regulated in cisplatin-resistant human lung adenocarcinoma A549/DDP cells compared with their parental cells [11]. Suppression of galectin-1 can sensitize lung cancer cells to cisplatin by up-regulating p38, mitogen-activated protein kinase, the extracellular signal-regulated kinase (ERK), and cyclooxygenase-2 [11,12] and down-regulating galectin-1 expression may promote sensitivity toward the proapoptotic and proautophagic drugs in glioma [27]. In contrast, increased expression of galectin-1 in epithelial ovarian cells can enhance their resistance to cisplatin by activating the H-Ras/Raf/ERK pathway and up-regulating the p21 and Bcl-2 [13]. Up-regulating galectin-1 in the hepatoma microenvironment by soluble galectin-1 during the cisplatin treatment could facilitate the chemoresistance of cancer cells via inducing autophagy [28]. In the present study, we found that the expression of galectin-1 was down-regulated both in cervical stromal cells and cervical cancer cells after chemotherapy, suggesting galectin-1 involved in chemotherapy response in cervical cancer. Moreover, patients with high levels of galectin-1 in stromal were more resistant to cisplatin-based NACT than those with low protein expression, which supported the recently highlighted roles of galectin-1 in the development of chemoresistance. It is likely that abundantly secreted galectin-1 proteins in stroma surrounding cervical tumors promoted tumor cell migration, invasion, and metastasis.

Integrin α5β1 is a glycoprotein adhesion molecule [14] and its alteration in many types of cancers related to the regulation of cell survival, proliferation, differentiation, apoptosis, and tumor angiogenesis [16,18,29]. Previous studies showed that integrin α5β1 was significantly increased in cervical cancer tissues compared with normal cervical mucosa, and higher levels of integrin α5β1 were related to poor histologic differentiation, lymph node metastasis, recurrence of cervical cancer, and unfavorable prognosis [19]. Consistent with this, our current study reported that mRNA levels of integrin α5 as well as integrin β1 were much higher in cervical cancer tissues than in normal cervical tissues.

Recently, some studies showed that integrin α5β1 had a close association with resistance to cancer chemotherapy. Nakahara et al. [17] reported oligosaccharide changes in integrin α5β1 were associated with cisplatin resistance in head and neck squamous cell carcinoma cell line. Hu et al. [30] suggested that overexpression of integrin α5 was strong risk factors for chemotherapeutic drug resistance in ovarian carcinoma patients. In agreement with previous studies, our data showed down-regulation of integrin α5β1 both in cancer cells and stromal cells following cisplatin-based NACT. Furthermore, we detected that integrin α5β1 expression in stromal was statistically lower in the chemotherapy response group than that in the non-response group, suggesting a higher level of integrin α5β1 in stromal may be a potential predictive biomarker for cisplatin chemoresitance in squamous cervical cancer patients.

Cisplatin-induced apoptosis is essential for the anticancer effect of cisplatin and inactivation of apoptosis may be the predominant cause for cisplatin resistance [23]. Caspases play critical roles in apoptosis. The activity of caspase 3, caspase 8, and caspase 9 was decreased in cisplatin-resistant cervical cancer cells [23]. In the present study, we found galectin-1 as well as integrin α5 levels were negatively correlated with caspase 3 and caspase 8 levels using TCGA RNA-sequencing data, suggesting that galectin-1 and integrin α5 may contribute to negative chemotherapy response via suppressing apoptosis.

In conclusion, our current study demonstrated that the expression of galectin-1 and integrin α5β1 in stromal can predict the responses to NACT in squamous cervical cancer. Further studies with larger cohorts are warranted to verify these results prospectively. It is also suggested from our findings that galectin-1 and integrin α5β1 may be implicated in the development of chemoresistance in cervical cancer via suppressing apoptosis. Our future study will further explore the role of galectin-1 and integrin α5β1 in chemoresistance *in vitro* and *in vivo.*

## Abbreviations

CR: complete response
ERK: the extracellular signal-regulated kinase
FIGO: International Federation of Gynecology and Obstetrics
GEO: Gene Expression Omnibus
NACT: neoadjuvant chemotherapy
PD: progressive disease
PR: partial response
SD: stable disease
TCGA: The Cancer Genome Atlas
